# Life after a point-of-care ultrasound course: setting up the right conditions!

**DOI:** 10.1186/s13089-020-00190-7

**Published:** 2020-09-07

**Authors:** T. J. Olgers, N. Azizi, H. R. Bouma, J. C. ter Maaten

**Affiliations:** 1 Dept Internal Med, Univ Groningen, Univ Med Ctr Groningen, 9700 RB Groningen, The Netherlands; 2 Dept of Clinical Pharmacy and Pharmacology, Univ Groningen, Univ Med Ctr Groningen, 9700 RB Groningen, The Netherlands

**Keywords:** POCUS, Point-of-care ultrasound, Ultrasound curriculum, Acute internal medicine, Ultrasound course, Barriers for ultrasound

## Abstract

**Background:**

Point-of-care Ultrasound (POCUS) is becoming an important diagnostic tool for internal medicine and ultrasound educational programs are being developed. An ultrasound course is often included in such a curriculum. We have performed a prospective observational questionnaire-based cohort study consisting of participants of a POCUS course for internal medicine in the Netherlands in a 2-year period. We investigated the usefulness of an ultrasound course and barriers participants encountered after the course.

**Results:**

55 participants (49%) completed the pre-course questionnaire, 29 (26%) completed the post-course questionnaire, 11 participants (10%) finalized the third questionnaire. The number of participants who performs POCUS was almost doubled after the course (from 34.5 to 65.5%). Almost all participants felt insufficiently skilled before the course which declined to 34.4% after the course. The majority (N = 26 [89.7%]) stated that this 2-day ultrasound course was sufficient enough to perform POCUS in daily practice but also changed daily practice. The most important barriers withholding them from performing ultrasound are lack of experts for supervision, insufficient practice time and absence of an ultrasound machine.

**Conclusions:**

This study shows that a 2-day hands-on ultrasound course seems a sufficient first step in an ultrasound curriculum for internal medicine physicians to obtain enough knowledge and skills to perform POCUS in clinical practice but it also changes clinical practice. However, there are barriers in the transfer to clinical practice that should be addressed which may improve curriculum designing.

## Background

Point-of-care Ultrasound (POCUS) is an important diagnostic tool for medical decision making and resuscitation of patients used by many specialists around the world. The reliability of the POCUS exam is largely operator dependent and, therefore, requires a solid ultrasound training system [1]. For internal medicine, it is a relatively new skill and ultrasound curricula are being developed to ensure correct use of POCUS for safe patient care [2–4]. In ultrasound curriculum designing, the first step is choosing the core applications which are relevant for that specific specialty and providing the core knowledge of these applications [2, 5]. This first step is called the initial introduction and usually consists of taking an ultrasound course, ranging from several hours to several days. The second step is gaining experience, followed by the third step which is achieving competency. If the initial introduction is not followed by the next, the usefulness of a course is questionable. Gaining experience by practicing under supervision is necessary to become competent, as is taking driving lessons after learning how to control a car. In our previous research, we have already shown that one important barrier for internal medicine residents to perform POCUS is the lack of experts to provide supervision [6]. To investigate the impact of an ultrasound course and the barriers to clinical implementation of POCUS after the course, we have performed a questionnaire-based study among ultrasound course participants.

## Methods

Since 2017, a 2-day ultrasound course specifically designed for internal medicine (DEUS course: Dutch Emergency UltraSound for internists) is being held 2–3 times a year. This is one of the two largest ultrasound courses for internal medicine in the Netherlands. These two courses are similar in content, educational tools, practice time and participants and both courses have a theoretical and practical exam. The number of participants in the DEUS course varies from 20 to a maximum of 24. We have invited all participants from 5 courses (*N* = 112), held in 2018 and 2019, to complete a questionnaire 1 week before the ultrasound course, 1–2 weeks after the ultrasound course, and 3 months after the course. The questionnaire included demographic data, questions about POCUS training and practice, and questions about the perceived usefulness and limitations. The survey was distributed by the course secretary board using an online survey tool (http://www.thesistoolpro.com). Participants were only invited once. Missing data were excluded from analysis for that specific questionnaire and for the returned questionnaires descriptive statistics was used. Participants provided written informed consent to participate in this study. SPSS statistics 23 was used for descriptive statistics.

## Results

55 participants (49%) completed the pre-course questionnaire, 29 (26%) completed the post-course questionnaire. Unfortunately, only 11 participants (10%) finalized the third questionnaire. Most participants were female (52.7%), and half of participants were aged between 30 and 39 (Table 1).

**Table 1:** Demographics of respondents

Demographics	Pre-course *N* = 55 (%)	Post-course 2 weeks *N* = 29 (%)	Post-course 3 months *N* = 11 (%)
Age			
18–29	13 (23.6)	6 (20.7)	2 (18.2)
30–39	27 (49.1)	15 (51.7)	6 (54.5)
40–49	8 (14.5)	5 (17.2)	2 (18.2)
50–59	7 (12.7)	3 (10.3)	1 (9.1)
Gender			
Male	26 (47.3)	12 (41.4)	4 (36.4)
Residency (year)			
1	5 (9.1)	1 (3.4)	2 (18.2)
2	5 (9.1)	4 (13.8)	2 (18.2)
3	13 (23.6)	6 (20.7)	1 (9.1)
4	4 (7.3)	2 (6.9)	0 (0)
5	2 (3.6)	1 (3.4)	1 (9.1)
6	2 (3.6)	2 (6.9)	0 (0)
None (student)	1 (1.8)	0 (0)	0 (0)
None (attending)	23 (41.8)	13 (44.9)	5 (45.4)
(Sub)specialty			
General internal medicine	19 (34.5)	12 (41.4)	5 (45.5)
Nephrology	4 (7.3)	3 (10.3)	1 (9.1)
Hemato-oncology	8 (14.5)	4 (13.8)	3 (27.3)
Acute internal medicine	13 (23.6)	4 (13.8)	1 (9.1)
Other	11 (20.0)	6 (20.7)	1 (9.1)
Workplace			
University hospital	14 (25.5)	9 (31.0)	5 (45.5)
Large Teaching hospital	37 (67.3)	19 (65.5)	4 (36.4)
Rural hospital	4 (7.3)	1 (3.4)	2 (18.2)
Ultrasound performed in hospital by			
Internists	21 (38.2)	12 (41.4)	5 (45.5)
Emergency physicians	46 (83.6)	25 (86.2)	7 (63.6)
US-machine available	49 (89.1)	25 (86.2)	8 (72.7)

Data presented as absolute number and percentage ()

The majority were internal medicine residents (56.4%), and a significant amount were attending internists (41.8%). The participants perceived that ultrasound was used by internists in their hospital in the minority (38.2%) of hospitals, in contrast to ultrasound use by emergency physicians (83.6%). The experience and practice of POCUS before and after the ultrasound course is shown in Table 2.

**Table 2:** Effect of ultrasound course on performing POCUS

	Pre-course *N *= 55 (%)	Post-course 2 weeks *N *= 29 (%)	Post-course 3 months *N *= 11 (%)
Performs POCUS			
No	36 (65.5)	9 (31.0)	2 (18.2)
Yes, mainly independent	8 (14.5	8 (27.6)	3 (27.3)
Yes, mainly supervised	11 (20.0)	12 (41.4)	6 (54.5)
Frequency of POCUS			
(Almost) daily	1 (1.8)	3 (10.3)	2 (18.2)
Multiple days a week	2 (3.6)	2 (6.9)	1 (9.1)
1 day a week	2 (3.6)	6 (20.7)	6 (54.5)
A few times per month	9 (16.4)	8 (27.6	2 (18.2)
(Almost) never	41 (74.5)	10 (34.5)	0 (0)

Number of POCUS performed^a^	Before course	After course	After course
0	36 (65.5)	9 (31.0)	2 (18.2)
1–20	12 (21.8)	20 (69.0)	7 (63.6)
21–60	4 (7.3)	0 (0)	2 (18.2)
61–100	2 (3.6)	0 (0)	0 (0)
101–200	0 (0)	0 (0)	0 (0)
> 200	1 (1.8)	0 (0)	0 (0)
Do you feel skilled			
No	46 (83.6)	1 (3.4)	3 (27.3)
Mostly not	8 (14.5)	9 (31.0)	3 (27.3)
Mostly yes	1 (1.8)	18 (62.1)	5 (45.5)
Yes	0 (0)	1 (3.4)	0 (0)
POCUS course sufficient to perform POCUS in daily practice?			
Yes	NA	26 (89.7)	10 (90.9)
No		3 (10.3)	1 (9.1)
Did following the course influenced your daily practice?			
Yes	NA	20 (69.0)	8 (72.7)
No		9 (31.0)	3 (27.3)

*POCUS* Point-of-care ultrasound, *NA* not applicable

^a^for post-course 2 weeks and post-course 3 months only number after the course; Data presented as absolute number and percentage ()

Most participants indicated they have never performed any ultrasound in clinical practice prior to the course (65.5%), which decreased after the course (34.5%). If they have performed POCUS after the course, it was largely done under supervision. After the course, more participants use POCUS and the number of POCUS performed also increased. This effect seems to persist after 3 months, notwithstanding the low number of respondents. Strikingly, nearly all participants (98%) indicated they were insufficiently skilled to perform POCUS in their own situation, but after the course this number decreased to 34.4%.

Of all post-course respondents, the majority stated that this 2-day ultrasound course was sufficient enough to perform POCUS in daily practice (Table 2). The majority also experienced that this ultrasound course influenced their clinical practice for various reasons. The most mentioned were that they felt more secure about performing ultrasound and they practiced ultrasound more, they have a better understanding of the value of POCUS for clinical decision making and POCUS increased their confidence of fluid status assessment. After 3 months, despite the low number of respondents, these percentages are roughly the same. Finally we have asked them for perceived barriers for performing POCUS (Table 3).

**Table 3:** Perceived barriers for performing POCUS

Perceived barriers	Pre-course *N* = 55 (%)	Post-course *N *= 29 (%)
Insufficient knowledge	33 (60.0)	2 (2.9)
Lack of adequate course	4 (7.3)	1 (3.4)
Insufficient experts/supervisors	22 (40.0)	17 (58.6)
Lack of US machine	12 (21.8)	13 (44.8)
Insufficient practice time	30 (54.5)	12 (41.4)
Insufficient opportunity for training under supervision	27 (49.1)	18 (62.1)
Resistance from other specialties	15 (27.3)	4 (13.8)
Lack of national guideline or from own profession	6 (10.9)	1 (3.4)
Other	3 (5.5)	3 (10.3)
None	3 (5.5)	0

Data presented as absolute number and percentage (); Post-course only including 2-week post-course

This shows that lack of knowledge is substantially reduced by following this ultrasound course. A significant amount of participants do not have access to an ultrasound machine. The majority were not able to practice under supervision due to lack of experts (58.6%) and/or practice time (41.4%). These two were perceived as the most important barriers to use POCUS.

## Discussion

We have shown that a 2-day hands-on ultrasound course is a solid first step in an ultrasound curriculum for internal medicine. Participants perceive they have sufficient knowledge and skills to perform POCUS in real practice, which is in line with the effect of other ultrasound courses and ultrasound curricula [7–10]. It should be noted that the number of post-course responders is lower as compared to pre-course responders, which possesses a high risk of bias. The second step in the curriculum, which is gaining experience, is difficult to fulfill if the conditions are not set properly. The largest remaining barriers in the Netherlands after following an ultrasound course, are lack of an ultrasound machine, lack of experts for supervision and lack of practice time. These barriers for learning POCUS are also seen in other specialties [11]. It is essential that we create a learning and training environment that is safe for patient care with enough experts available for supervision. Skills including ultrasound skills may subside quickly if ultrasound is not performed on a regular basis, especially by novel sonographers [12–14]. This implies that before an ultrasound course is offered as a start for the ultrasound curriculum, the conditions should be optimized for participants to complete all necessary steps in achieving and maintaining competency and not only the initial introduction [15, 16]. For example in the Netherlands, POCUS has been made a mandatory skill for internal medicine residents by the Dutch Internal Medicine Federation (NIV). This obliged educators and hospitals to include ultrasound machines in their budget and to create focus groups for further development of POCUS educational programs. However, with increasing amounts of affordable handheld devices with good quality images, budget problems may become less of an issue. It required investing time and money to educate and train experts in the teaching hospitals for supervision of residents and to guarantee safe patient care. Addressing these issues are at least as important as sending staff or residents to an ultrasound course. In this way an ultrasound courses will not only be just a pleasant experience but a real start of an ultrasound curriculum.

### Limitations

This survey has some potential limitations. The first questionnaire response rate was 49%, the post-course survey 26% and the 3-month response rate was only 10%. Most web-based data collection have a mean response rate of 27.6% [17]. The low response rate may have influenced the interpretation of the results. Especially the 3^rd^ questionnaire must be seen as indicative due to the low number of respondents. Theoretically, if all post-course non-responders represent participants that did not benefit from the course in terms of knowledge, the number of participants with insufficient knowledge may have only decreased from 60 to 51%, although this is very unlikely. In addition, responders may be more enthusiastic about ultrasound and can have different ideas than non-responders. Furthermore, responders may feel more confident about their knowledge after a course than non-responders which may have influenced the low percentage of the barrier lack-of-knowledge. Finally, participants may overestimate their own knowledge, a well-known psychological phenomena in unskilled participants called the Dunning–Kruger effect, although we do not know if this is applicable for our study population [18]. We think an ultrasound course might even have the opposite effect if participants have become more aware of their shortcomings (conscious incompetence) and underestimate their skills. We have shown an increase in their perceived knowledge. How this course actually increase ultrasound skills was beyond the scope of this research as we only asked participants for their perceived skills. Half of the respondents were residents and half were attending physicians, there also was a variability in their corresponding internal subspecialties but selection bias cannot be excluded. We did not contact a subgroup of non-responders nor did we sent structured reminders to complete the survey which could have increased the response rate. We omitted open questions on most topics so specific important issues not addressed in our questionnaire may be missed. This research represents the course participants perceived skills and knowledge but we did not test them afterwards for ultrasound skills. We only invited participants of one of the two ultrasound courses. These courses are very similar and faculty of both courses are also members of the Dutch taskforce developing the ultrasound curriculum in the Netherlands and some teach in both courses. We would expect the same results if we invited all participants.

## Conclusion

This study shows that a 2-day hands-on ultrasound course is a sufficient first step in an ultrasound curriculum for internal medicine physicians. However, the conditions should be optimized to prevent loss of this knowledge and skills so course participants can proceed to the necessary consecutive steps which are gaining experience and achieving competency. The most important barriers to overcome are lack of experts for supervision and insufficient practice time, both problems should be addressed before attending an ultrasound course to increase the yield.

## Data Availability

The datasets used and/or analyzed during the current study are available from the corresponding author on reasonable request.
